# Simple, Fast, and Reliable Analysis of Label‐Free Proteomics Data With the Proteomics Eye (ProtE)

**DOI:** 10.1002/prca.70037

**Published:** 2025-12-26

**Authors:** Theodoros Margelos, Manousos Makridakis, Charis Gonidaki, Foteini Paradeisi, Manos Vossos, Jerome Zoidakis, Antonia Vlahou, Rafael Stroggilos

**Affiliations:** ^1^ Center of Systems Biology Biomedical Research Foundation Academy of Athens Athens Greece; ^2^ Department of Applied Mathematics, Faculty of Electrical Engineering Mathematics and Computer Science University of Twente Enschede the Netherlands; ^3^ Department of Biochemistry and Molecular Biology, Faculty of Biology National and Kapodistrian University of Athens Athens Greece

**Keywords:** bioinformatics, computational proteomics, DIANN, MaxQuant, Proteome Discoverer

## Abstract

In label‐free mass spectrometry experiments, the data output is typically a proteome table that requires further processing, quality testing, and visualization to fully interpret the captured proteomic signals. Currently, post‐quantification analysis of these tables often relies on complex programmatic pipelines, which can become challenging to use. Here, we introduce the Proteomics Eye (ProtE), a single‐function R package designed to streamline the analysis of proteome tables generated by commonly used software tools (DIA‐NN, ProteomeDiscoverer, and MaxQuant). ProtE provides a broad range of options for data processing, preparation, and statistical testing. It also performs gene set enrichment analysis and offers a comprehensive suite of visualization plots to assess data quality and facilitate biological interpretation. Given a categorical variable with two or more groups, ProtE enables group‐wide and pairwise statistical comparisons across all group combinations, using both traditional statistical tests and linear models for differential expression analysis. By integrating all these features into a single, user‐friendly R function, ProtE simplifies the analysis of large‐scale label‐free DDA and DIA datasets, making advanced proteomic analysis accessible to both experienced researchers and beginners.

1

The emergence of quantitative proteomics in the last two decades has led to a breakthrough in biological science research [1]. It has enhanced the discovery of novel biomarkers and drug development [2, 3], the study of disease progression [4], and boosted the understanding of cellular signaling mechanisms [5]. This was enabled by the advancement of mass spectrometry (MS) technologies, which allowed not only the identification of up to thousands of proteins per sample but also their robust quantification [6]. Optimized data acquisition methods such as Data Dependent (DDA) and Data Independent Acquisition (DIA) [7] in combination with label‐free or labeling methods for protein quantification produce “rich” results; and various software tools have been developed for respective data processing and further analysis [8]. Such tools map the spectrometric data to peptide identifications and have internal methods for peptide or proteome‐level quantification. With the prolific expansion of bioinformatics, new data processing methods have been introduced to the field of MS, increasing the heterogeneity of the downstream proteomics analysis workflow [9]. Navigating between the different options and building the right workflow for analyzing proteomics data can become challenging even for experienced researchers.

Various tools have been developed toward standardizing the analysis of proteome‐level data. These tools have been primarily established as R packages, Python libraries, or Shiny‐based websites. Available solutions like the Proteus software, MSstats [10], protti [11], MSnbase [12], NormalyzerDE [13], prolfqua [14], tidyProteomics [15], pyOpenMS [16], MSPypeline [17], AlphaPeptStats [18], OmicScope [19], F1000Research workflows [20], and MS‐DAP [21] offer extensive functionality for either data manipulation or statistical analysis. While each has unique advantages, they often require significant bioinformatics expertise, as their bottom‐up workflows rely on multiple functions and complex input transformations. Furthermore, for differential expression analysis, they mostly employ only linear model statistics and do not employ nonparametric tests. Website tools like ProtExA [22], EasyPubPlot [23], ProteoSign [24], and PIQMIe [25] are easier to utilize, enabling users with no programming skills to perform analyses. However, these platforms raise concerns with respect to data privacy and security, while they are dependent on stable internet access and functional servers.

As proteomics experiments are frequently designed by researchers in non‐proteomics laboratories, it is common for individuals with little or no programming expertise to end up with large proteome tables that are complexand time‐consuming to process and analyze. On the other hand, proteomics facilities are often constrained by high workloads, and thus, they cannot afford to allocate extended resources for analytical data tasks. To address these challenges, we introduce the *Proteomics Eye* (ProtE), an R package that automates, standardizes, and accelerates the label‐free proteomics workflow in a user‐friendly way (Figure 1).

**FIGURE 1:**
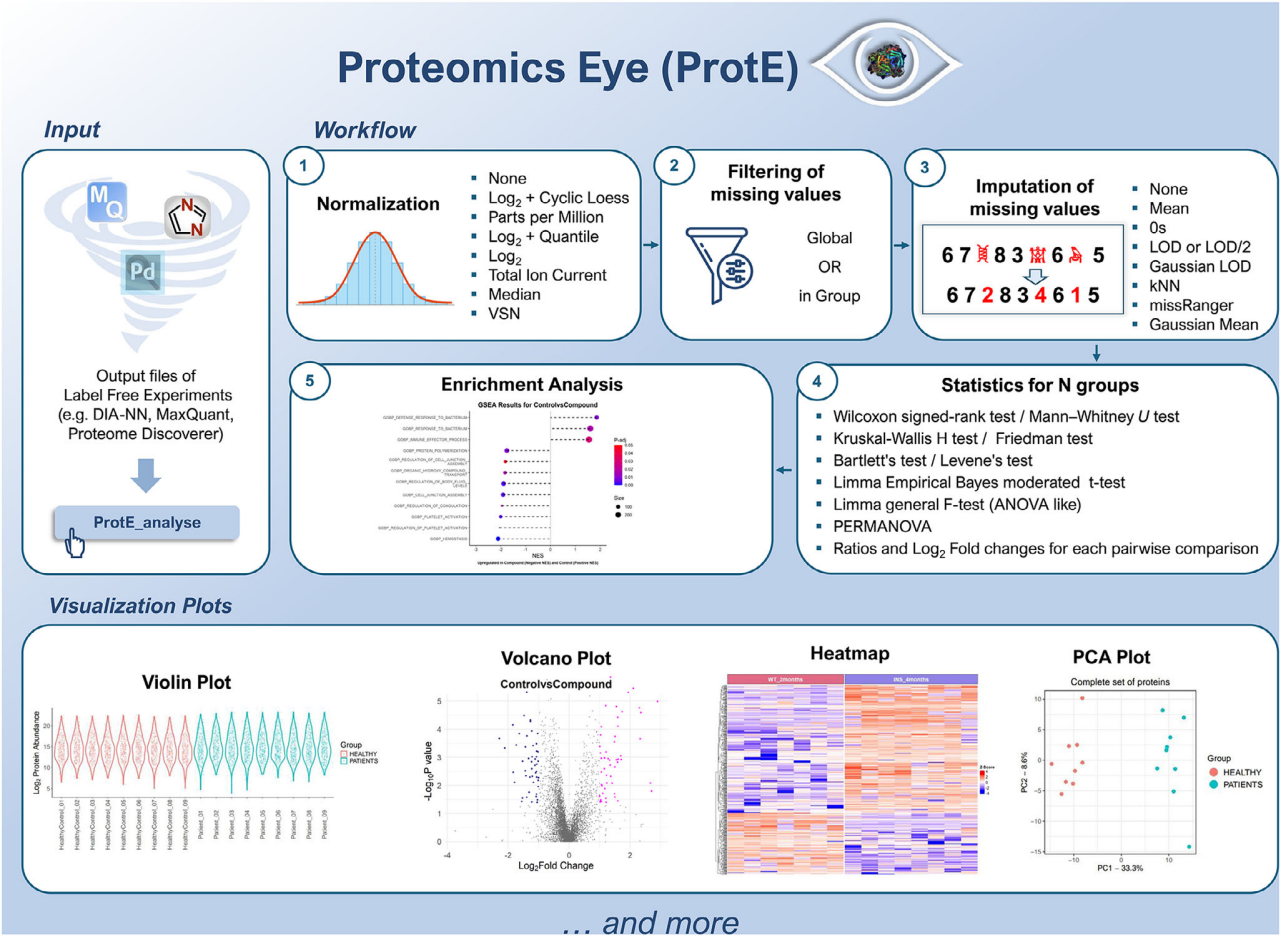
The Proteomics Eye (ProtE) pipeline. The figure illustrates the type of input files that can be parsed to the ProtE_analyse function, the data processing workflow with the different options for normalization, filtering of missing values, imputation, as well as the statistical output, and the enrichment analysis together with some of the visualization plots produced by ProtE.

ProtE stands out for its unique ability to wrap the entire post‐quantification workflow in a single function call, ProtE_analyse(), executable in a matter of minutes or even seconds. Simultaneously, it offers users the flexibility to input independent or paired experimental groups and analyze them using both traditional non‐parametric statistical tests alongside linear models, which can incorporate fixed effects as covariates, while saving the results directly on the user's PC.

ProtE is tailored for the analysis of proteomics data quantified by the commonly used spectral processing tools, Proteome Discoverer (Thermo Fisher Scientific, Waltham, USA), MaxQuant [26], and DIA‐NN [27] (or FragPipe's‐DIA‐NN output). We designed ProtE from the perspective of the user having little or no programming expertise. Given an independent categorical variable with *N* groups, ProtE unites in just one function call all necessary processing steps together with the ability to perform all possible N*(*N*−1)/2 pairwise statistical comparisons. This makes ProtE highly versatile, freeing users from the necessity to subset data or manually iterate analyses, significantly reducing the effort and potential for error in downstream analysis. In brief, ProtE runs the following steps in the depicted order:

1. Fetching UniProt information if a column “Description” or “Fasta Description” is not included in the input data table
2. Visualization of the data prior to any processing
3. Normalization
4. Filtering of frequently missing proteins
5. Imputation
6. Visualization of the processed data
7. Statistical analysis
8. Visualization of statistical output in the form of plots
9. Enrichment analysis

The main function of ProtE, ProtE_analyse(), reads, processes, and analyzes proteome tables generated by Proteome Discoverer, MaxQuant, DIA‐NN, or Frag‐Pipe DIA‐NN (Table 1).

**TABLE 1:** Information about the proteome tables that can be parsed and analyzed by ProtE's main function ProtE_analyse().

Use cases	Software tool	Input for function
1.	DIA‐NN (or DIA‐NN output from FragPipe) in .tsv or .xlsx format	Table with all samples (unique_genes_matrix or pg_matrix files)
2.	MaxQuant (ProteinGroups file in .txt or .xlsx format)	Table with all samples ProteinGroups file
3.	Proteome Discoverer (one table with all samples)	Table with all samples (.xlsx or .txt file)
4.	Proteome Discoverer (one .xlsx or .txt file per sample, usually exported from .MSF files)	Group folders with .xlsx or .txt files (one per sample), provide via *pd_single_dir* argument.

ProtE runs in two modes, depending on the existence of sample metadata. If a metadata file is available, ProtE will incorporate all the metadata variables into the statistical model, provided they do not contain any missing values (a single missing value will drop the given variable out of the model). The metadata file needs to be organized in the following simple way: the first column must contain the sample names written exactly as they appear in the proteomics data, while the second column should contain the independent variable that formulates the main groups of the experiment. Every next column of the metadata can contain covariates whose inclusion in the model will affect the statistical results of the independent variable.

If a metadata file is not available, ProtE still supports statistical testing of the independent variable, as long as the samples in the input proteome table are positioned consecutively to each other according to their experimental group (the details on the underlying data structure are provided in the Supporting File). Users can then define the sample‐to‐group labeling and the corresponding group sizes by using the function's parameters *group_names = c()*, and *samples_per_group = c()*, respectively (Figure 2A(ii)).

**FIGURE 2:**
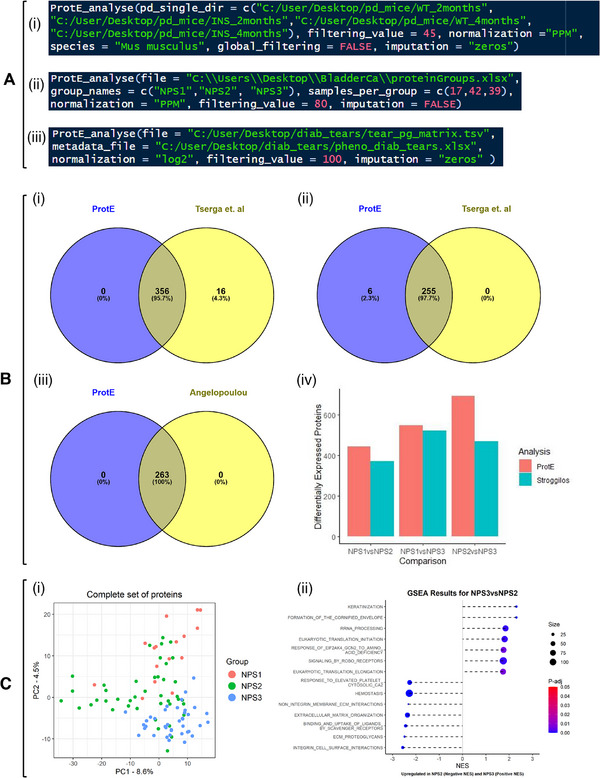
(A) (i, ii, iii): the lines of code of the ProtE_analyse function for the ProtE run, for Tserga's, Angelopoulou's, and Stroggilos's data, respectively. Note that for (i),(iii), a metadata file was supplied, while for (ii), the information about the groups of comparison was provided inside the function. (B) Venn diagram showing the number of commonly identified differentially expressed proteins (DEPs) between Tserga's and ProtE's analyses, between Ins2Akita and Wt mice aged 2 (i) and 4 (ii) months old. (ii) Venn diagram showing that ProtE's analysis replicated Angelopoulou's analysis for diabetic vs. healthy tears. (iv) Barplot showing the DEPs found in the non‐muscle invasive proteomic subtype (NPS) comparisons from Stroggilos, when ran with the MaxQuant output file. (C) Visualization plots of the Principal Component Analysis (PCA) (i) and the Gene Set Enrichment Analysis (ii) that are also performed in every ProtE_analyse run.

The power of ProtE as an analytical tool lies not only in its simplicity but also in its rich arsenal of data processing and preparation options, all defined as parameters of the same R function. A standard step in proteomics workflows is the normalization of raw data, which adjusts intensity values to mitigate technical biases and systematic errors, enabling more reliable biological comparison [28]. ProtE allows users to choose from seven distinct normalization methods to address potential technical biases. Another common challenge of mass spectrometry proteomics data is the prevalence of missing values (MVs) in the quantitative measurements: These (MVs) arise from proteins not quantified by spectral analysis tools, often representing 10%–50% of proteins per sample [30]. ProtE allows users to filter out proteins exceeding a user‐defined MV threshold, applied either across all samples or separately within experimental groups. Following this protein filtering, imputation is performed, wherein MVs are substituted with plausible quantitative values. ProtE provides eight imputation methods, optimized for various scenarios. All processing options (Figure 1) are detailed in the Supporting File and at https://github.com/theomargel/ProtE.

These analytical parameters are explicitly chosen by the user through the ProtE_analyse() function. For example, users can select filtering, normalization, and imputation methods by filling the respective parameters inside the function call. By adjusting one or more of these parameters and rerunning the function, ProtE enables users to directly assess how different data processing choices influence statistical results. A summary report is generated at the end of each analysis, which details all transformations applied to the data for the specific ProtE_analyse() run, (e.g., specific normalization, imputation methods, and missing values frequency thresholds used).

Following data processing, ProtE performs statistical tests and differential expression analysis, to determine proteins with significantly different abundances between the experimental groups under comparison. An option unique to ProtE, is that users can specify whether this analysis will be performed on independent or paired samples (samples from the same individual/cell/population but, e.g., from different timepoints), via the parameter “independent,” which can be respectively set to TRUE or FALSE. ProtE generates lists of differentially expressed proteins using both nonparametric tests and linear models. The statistical output of the former is stored in the Excel file traditional_statistics.xlsx and includes Wilcoxon's test results of pairwise comparisons, Kruskal–Wallis or Friedman's test results when groups are more than two, and PERMANOVA pseudo‐F, R^2^, and *p* value for the complete feature set, via the package vegan [29]. For assessing the equality of the group variances (homoscedasticity), ProtE implements Levene's and Bartlett's statistical tests. In all cases, the average abundance of each protein per group, ratio of the means and the corresponding log_2_‐fold change values, nominal *p* values, and false discovery rates (method of adjusting the *p* values for multiple hypotheses manually selected by the user) per comparison are provided.

For linear model‐based statistics, ProtE leverages the limma package, applying log_2_ transformation, and Bayesian regression to the data, before fitting them to a linear model, with results saved in the ‘dataset_limma_test.xlsx’ file [31]. These include coefficients for each experimental group and any covariates (e.g., age, batch effects) incorporated into the model. For paired sample experiments, subjects are treated as random effects in the linear model using a blocking approach, while any covariates are incorporated into the design matrix of the model [32]. Specifically, the output features result from an ANOVA‐like *F*‐test for variance comparison when there are more than two groups, alongside results from moderated *t*‐tests for each pairwise comparison, and log_2_ fold changes of the moderated data.

By further utilizing the package fgsea [33], ProtE performs gene set enrichment analysis, to determine the degree of association of the differentially expressed proteins with specific gene sets or gene ontology terms. This analysis is conducted using as metric score, the log_2_ fold changes of the pairwise comparisons. The user can select a collection from the MSigDB [34] via the arguments subcollection and species, with options including the commonly used Reactome [35] and Gene Ontology [36] gene sets. The results are saved inside an Excel file named GSEA_results.xlsx that contains the enriched pathways of each pairwise comparison in different Excel sheets.

To enable evaluation of data quality and to enhance biological interpretation, ProtE offers a comprehensive suite of visualization means common to proteomic datasets. This encompasses violin plots and boxplots of the protein abundances in each sample and a mean‐standard deviation relation plot before and after the processing of the data, to assess the effects of normalization and imputation. Also, a log_2_ abundance rank plot, a histogram of the imputed values (in case multiple values imputation has been selected), Variation of Coefficient plots, and PCA plots considering both the full dataset and the significantly altered proteins are created. Heatmaps, which illustrate abundance alterations across experimental groups, as well as volcano plots are generated. Finally, plots displaying the most enriched, significant pathways between each contrast are also created. All plots are saved in BMP format, and they are named automatically based on what they are displaying, After_Processing_Boxplot.bmp, for example is the boxplot of the protein intensities of each sample after data processing (e.g., normalization, filtering, and imputation). Additionally, output messages in the RStudio console will explicitly state the content of the output files.

As ProtE offers various methods for data processing and visualization, users can iterate multiple runs of the function with different parameters assigned to each run and then assess which method best fits their data. This iterative approach is particularly useful for tuning thresholds like missing value filtering or imputation strategies, allowing the comparison of the QC plots and the statistical outputs to optimize for biological relevance while minimizing technical artifacts. This way, users can systematically refine their pipeline without using repetitive lines of code.

To demonstrate the capabilities of ProtE, published proteomics data derived from Proteome Discoverer, DIA‐NN, and MaxQuant, respectively, were analyzed using the ProtE_analyse function.

Specifically, proteomics data from Tserga et al., which compared the proteomics profiles of Ins2Akita type I diabetic mice and matched wild‐type controls across two time points (2 and 4 months old), were analyzed [37]. The analysis identified 255 DEPs between 2‐month‐old Ins2Akita and wild‐type mice and 372 DEPs between 4‐month‐old Ins2Akita and wild‐type mice, based on a Mann–Whitney *p* value threshold of <0.05. The processed data, available in the Supporting File of the paper, were generated using Proteome Discoverer 1.4 with the SEQUEST search engine and the UniProt mouse (Mus musculus) database, exported as one file per sample. After organizing the files for each experimental group (Ins2Akita and wild‐type) into distinct folders, the respective directories were used as input into ProtE_analyse alongside post‐processing options according to the original publication: Parts per Million (PPM) normalization, filtering value set to 45 (which means that proteins with less than 45% missing values across samples are kept), imputation of missing values as zeros. ProtE identified 261 DEPs (Mann–Whitney *p* value <0.05) for the 2‐month‐old mice, encompassing all 255 DEPs reported by Tserga et al. (Figure 2B(i)) and 356 DEPs for the 4‐month‐old mice, all of which were included in the initial analysis (Figure 2B(ii)).

Additionally, proteomics data from the PRIDE database (Project PXD052994) were parsed, derived from the study by Angelopoulou et al. [38]. This study compared the tear proteomics profiles of children with type I diabetes to those of age‐matched healthy controls. The raw mass spectrometry data were processed as described [38] using DIA‐NN (version 1.8.1) and searched against the Homo sapiens UniProt database. Their analysis identified 263 DEPs between diabetic and healthy subjects, based on a Mann–Whitney *p* value threshold of <0.05. The DIA‐NN pg_matrix output file was provided to ProtE, and post‐processing steps were replicated as in the original analysis (log_2_ transformation as normalization, filtering value set to 100%, e.g., no filtering is applied, and imputation of missing values as 0). Using ProtE with these parameters, all 263 DEPs (Mann–Whitney *p* value <0.05) reported in the original publication were identified (Figure 2B(iii)).

As a further showcase of the interoperability of ProtE between Proteome Discoverer and MaxQuant quantified proteomes, a re‐analysis of data from Stroggilos et al. (2019) was also performed [39]. In the original study, label‐free DDA raw files from a total of 98 tissue samples were analyzed by Proteome Discoverer, and the authors identified three proteomic subtypes (NPS1, NPS2, and NPS3), which were further tested for statistically significant differences in clinical and proteomic features. We sought to investigate whether ProtE could recapitulate the statistical analysis of the three subtypes, following switching the quantification software from Proteome Discoverer to MaxQuant. The 98 raw files were processed with MaxQuant (v2.6.5) using the same instrument parameters as in the original publication, and the output file, “proteinGroups.txt,” was subsequently provided to ProtE. The same post‐processing options were applied: raw intensity values were normalized to the parts‐per‐million (PPM) scale, per‐protein missing values were calculated globally across all 98 samples, proteins with missing values in more than 80% of samples were excluded, and imputation was disabled (imputation parameter set to FALSE). Figure 2B(iv) illustrates the DEPs identified (Mann–Whitney *p* value <0.05) in the original analysis compared to those identified using ProtE_analyse. Slight differences are expected due to the use of different spectral data search engines in each of Proteome Discoverer and MaxQuant.

Overall, based on the above‐mentioned test cases, the ProtE_analyse function closely approximates the findings of the original publications, demonstrating ProtE's ability to replicate analyses and deliver reliable results. To highlight ProtE's efficiency, the analysis of each of the three datasets was performed with a few lines of code, as shown in Figure 2A, and completed within 2 min.

While ProtE is built upon established methods, such as known normalization techniques (e.g., log2, VSN), imputation strategies (e.g., kNN, missRanger), or widely used statistical tests (e.g., limma, Kruskal–Wallis), its primary contribution lies in integrating these methods into a single, user‐friendly function, ProtE_analyse(). ProtE addresses the gap of requiring bioinformatics expertise to perform a proteomics analysis, by automating the entire analysis pipeline—from data processing to statistical analysis and visualization—making it accessible to a broader audience. Its instant acceptance of multiple input formats (e.g., MaxQuant, DIA‐NN, and Proteome Discoverer) further enhances its utility, compared to different tools where data tables need to be previously edited.  Its efficacy is also highlighted by the inclusion of other statistical tests such as the option for paired analysis, the inclusion of both traditional tests and linear models, or additional statistical tests like PERMANOVA and Levene's test for assessing the equality of variances, and gene set enrichment analyses that are not included in other proteomics analysis tools. This combination of accessibility, comprehensiveness, and flexibility constitutes a meaningful advancement, even if ProtE does not introduce entirely new algorithms.

As limitations, the current version of ProtE is designed to analyze data already summarized at the protein level, rather than incorporating peptide‐level intensity data, and thus cannot influence decisions made at the pre‐quantification stage. Its differential analysis methods are optimized for straightforward experimental designs with categorical variables as groups of comparison, and they do not yet support complex experiments involving interactions between multiple factors and continuous variables as groups. These limitations highlight opportunities for expansion. In future versions, we aim to enhance ProtE by integrating peptide‐to‐protein summarization and extending its statistical capabilities to accommodate a broader range of experimental designs.

To conclude, ProtE is a new R package tailored for a wide range of users, from those with no bioinformatics background to advanced researchers. It performs proteomic analysis in the output data in a straightforward, easy‐to‐handle pipeline, in just one function call. As large‐scale proteomics data are accumulated, ProtE fulfills the need for quick, reliable, and standardized processing and analysis. We invite the proteomics community to experiment with ProtE and freely provide any feedback via the GitHub repo: https://github.com/theomargel/ProtE/. The GitHub site includes installation instructions as well as a complete guide for the function, which are also presented in the Supporting File.

## Funding

The authors have nothing to report.

## Conflicts of Interest

The authors declare no conflicts of interest.

## Supporting information




**Supporting Information File 1:** prca70037‐sup‐0001‐SuppMat.pdf.

## Data Availability

No new data were generated or analyzed in this study. All data supporting the findings were obtained from publicly available datasets in Tserga et al. (2024) [DOI: 10.3390/ijms25031387], Angelopoulou et al. (2024) via PRIDE (PXD052994), and Stroggilos et al. (2019) [DOI: 10.1002/ijc.32556], as cited in the reference list.
