# Supplementary material for: Simple, Fast, and Reliable Analysis of Label‐Free Proteomics Data With the Proteomics Eye (ProtE)
Source: Proteomics Clin Appl. 2025 Dec 26;20(1):e70037. doi: 10.1002/prca.70037 (PMC12743176; doi:10.1002/prca.70037)
Supplement: Supplementary file 1 — Supporting Information File 1: prca70037‐sup‐0001‐SuppMat.pdf. [file PRCA-20-e70037-s001.pdf]

ProtE manual guide

Installation instructions

If you have already installed both the R Studio environment and the Rtools, skip to Step 4.

Step 1: Install R

To get started with R, first download and install the latest version of R from the official CRAN website:

- Go to the R download page.
- Select the appropriate version for your operating system:
  - **Windows**: Click on "Download R for Windows".
  - **MacOS**: Click on "Download R for macOS".
  - **Linux**: Follow the instructions based on your distribution.

Once downloaded, run the installer and follow the instructions to complete the installation.

Step 2: Install RStudio

Next, you will need an Integrated Development Environment (IDE) to work with R. The most popular IDE is RStudio.

- Go to the RStudio download page.
- Select "RStudio Desktop" and download the installer for your operating system.

Run the installer and follow the on-screen instructions to install RStudio.

Step 3: Install RTools (For Windows Users)

RTools is necessary if you need to compile packages from source on Windows, which is common when installing certain R packages.

- Go to the RTools download page.
- Download the version of RTools that matches your R version.
- Run the installer and follow the instructions.

Make sure to select the option that allows RTools to be added to your system path during installation (i.e., save the program to ProgramFiles)

Step 4: Download the Package

Install the development version of ProtE from GitHub:

```
if (!require("pak", quietly = TRUE)) {
      install.packages("pak",binary = TRUE)}

    pak::pak("theomargel/ProtE", dependencies = TRUE)
```

Then load its library with:

```
library(ProtE)
```

Note that when ProtE_analyse() function runs for the first time, the msigdbdf package will be downloaded, which will take up to some seconds. Whether you are asked, if you permit the installation of msigdbdf select: **Yes**.

ProtE_analyse()

The entire proteomics processing and analysis workflow is performed using the *ProtE_analyse()* function. This function is designed to work with a proteome table where protein IDs populate the rows, and experimental samples populate the columns. ProtE imposes no restrictions on the number of groups a user wishes to analyze. Users can theoretically provide an unlimited number of folder paths, and the function will automatically perform statistical analysis for every possible pairwise group comparison.

Input files

ProtE_analyse() accepts as input, files generated from Label-Free Proteomics workflows. These input files are produced by three widely used Mass Spectrometry data analysis tools: Proteome Discoverer, MaxQuant and DIA-NN (or the DIA-NN output generated by FragPipe)

Use Cases	Software tool	Input for function
1.	DIA-NN (or DIA-NN output from FragPipe) in .tsv or .xlsx format	Table with all samples (unique_genes_matrix or pg_matrix files)
2.	Proteome Discoverer (one table with all samples)	Table with all samples (.xlsx or .txt file)
3.	MaxQuant (ProteinGroups file in .txt or .xlsx format)	Table with all samples ProteinGroups file
4.	Proteome Discoverer (one .txt or .xlsx file per sample, usually exported from .msf files)	Group folders with .xlsx files (one per sample), parse via *pd_single_dir* argument.

Table 1. Information about which input files can be input in function ProtE_analyse()

The input files for cases 1-3 should be fed in the parameter *file,* while for use case 4 the path to the folder(s) which contain the .xlsx files for each sample, should be parsed in the parameter *pd_single_dir*.

A metadata table (.xlsx or .txt format) is **optional** to be provided, via the parameter *metadata_file*. It must contain the names of the samples that should match the naming inside the input table (or the naming of the Excel files for use case 4.) in the 1st column, and the experimental Groups of comparison in the 2nd column. The next columns can include information about covariates that will influence the statistical analysis.

	A	B	C	D	E	F
1	**PatientID**	**Consensus Subtype**	**Gender**	**Stage**	**Grade**	**Squamous differentiation**
2	Sample8	NPS1	Male	T1	3	Yes
3	Sample12	NPS1	Male	T1	3	No
4	Sample20	NPS2	Male	T1	3	No
5	Sample25	NPS2	Female	T1	3	No
6	Sample29	NPS2	Male	T1	3	No
7	Sample1	NPS1	Male	T1	3	No
8	Sample4	NPS1	Male	T1	3	No
9	Sample6	NPS1	Male	T1	1	Yes
10	Sample9	NPS1	Male	T1	3	No
11	Sample27	NPS2	Male	T1	1	No
12	Sample28	NPS2	Male	T1	3	Yes
13	Sample30	NPS2	Male	T1	1	Yes
14	Sample32	NPS2	Male	T1	1	No
15	Sample60	NPS3	Male	T1	1	No
16	Sample62	NPS3	Male	T1	1	No
17	Sample63	NPS3	Male	T1	1	No
18	Sample64	NPS3	Male	T1	1	No
19	Sample65	NPS3	Female	T1	1	No
20	Sample66	NPS3	Male	T1	1	No
21	Sample2	NPS1	Male	T1	3	No
22	Sample3	NPS1	Female	T1	3	No
23	Sample5	NPS1	Male	T1	3	Yes
24	Sample7	NPS1	Male	T1	3	No
25	Sample10	NPS1	Male	T1	3	No
26	Sample11	NPS1	Male	T1	2	Yes

Figure 1. An example of the metadata file that can be inserted in the *ProtE_analyse()* function. The first column should contain the names of the samples, the second column includes the experimental groups of comparison (for instance NPS1, NPS2, NPS3) while

ProtE

the rest of the columns should include covariates that will be taken into account for the limma statistical analysis (e.g. Gender, Stage etc.).

Assuming a MaxQuant quantification has been performed, the file ProteinGroups.txt (or ProteinGroups.xlsx) shall be inserted to ProtE_analyse's *file* parameter. To copy-paste the file path in Windows, locate the desired file inside your folders and right-click the file, then select "Copy as Path" from the context menu. Go to RStudio and paste the path. Because usually the directories will be separated with a single backlash, ensure to use forward slashes (/) for specifying paths or adding a second backlash.

e.g:

```
ProtE_analyse(file = "C:\\Bioprojects\\BreastCancer\\Proteomics\\MaxQuant\\ProteinGroups.txt")
```

or

```
ProtE_analyse(file = "C:/Bioprojects/BreastCancer/Proteomics/MaxQuant/ProteinGroups.txt")
```

The same logic for the directory separators slashes applies when inserting the *metadata_file* and *pd_single_dir* parameters. If the user provides a metadata file, the multiple files can be saved inside one folder, who will be provided via the parameter *pd_single_dir* .

e.g.:

```
ProtE_analyse(pd_single_dir = "C:\\bCa\\BreastCancer\\Proteomics\\ProteomeDiscoverer",
metadata_file = "C:\\Bioprojects\\bCa\\Proteomics\\ProteomeDiscoverer\\metadata.xlsx")
```

ProtE_analyse() detects the sample columns in the proteome table automatically, either based on a key word or based on the expected structure of the table. For ProteomeDiscoverer it expects a table (.xlsx or .txt) File generated by Proteome Discoverer (usually from the .pdResult object) and detects sample columns based on their column names, which must contain the word "Abundance", or single files per sample (usually from .msf Excel exports). For MaxQuant it accepts as input the file proteinGroups.txt and detects sample columns based on the columns with "Intensity " or "LFQ intensity" (note the space character after the word Intensity, which is used to differentiate the actual samples from the column named "Intensity", the latter containing the pooled intensity of all samples), while for DIA-NN outputs it accepts either the unique_genes_matrix.tsv or the pg_matrix.tsv files or the same tables converted to Excel file format, and detects the sample columns based on the structure of the data.

Analysis when a metadata_file is not inserted:

For use cases 1-3 (Table 1) users need to rearrange the columns of their proteome tables so that samples belonging to the same group are positioned next to each other, as shown in **Figure 2**. Note that in this case, the user should also fill the parameters *group_names* and *samples_per_group* .

Control_01	Control_02	Control_03	Control_04	Control_05	Treatment_01	Treatment_02	Treatment_03	Treatment_04	Treatment_05	Compound_01	Compound_02	Compound_01	Compound_03	Compound_04	Compound_05
32383.7	30537.2	67524.4	89304	35435.9	26684.3	53772.8	91589.2	117898	24211.9	22461.8	42344.3	30208.3	41789.1	27129.8	11829.2
71841.4	11824.5	39618.4	22357.8	110701	12974.6	28489.5	22172.1	22074.3	89694.1	90052.5	90067.9	22822.3	48905.7	78093.4	39410.4
14035.8	3244.62	17291.1	5798.96	11073	3531.31	12769	6688.24	10031.8	14530.7		13018.8	5695.38	15854.5	10793.8	6775.72
14988.8	5835.55	17783.1	4572.13	23713.7	5200.05	19676.9	15718.4	14468.5	21109	13051.5	21337.8	7516.03	19901.9	22832.1	9330.06
11777.9	5503.66	17466.8	9289.45	13005.7	3893.95	8801.44	3267.41	13272	21993.7	72564.4	25733.2	5383.41	7898.91	23443.9	10515.2
									15218.9			11990.6			
8171.47	12096.1	15440.3	4966.21	12963.7		8027.92	8401.36	12325.6	16333.6	6224.25	7983.8	18124	8808.54	4383.08	7818.69
9032.2	8064.13	6490.02	7814.07	11721.7	7945.44	13148.6	10331.4	4641.5	12707	12076.2	18960	9801.13	17579.4	21290.2	8882.03
438.969	691.713	531.347	280.176	489.564		1410.15			2380.34	5000.03	188.479	426.85		1363.26	
3026.28	1907.77	2863.44	907.231	1918.75	1893.7	4155.68	4447.39	390.327	41056.8	18596.5	27430.6	65051.6	36075.3	26810.6	25857
5299.79	5094.58	4223.51	2525.54	10199.7	1188.12	3246.17	3180.79	3298.62	15409.9	2597.67	6385.9	9522.3	4800.67	4321.86	6631.11
47717.4	54908.5	70602.4	34435.4	29463.7	33798.4	36691.6	44331.2	27272.5	33561.9	25486.6	41939.6	71681.2	62829	24786.9	20783.4
4254.27	5538.27		4599.85	2931.92		1545.71	2611.21		34505.2	46716.2	60087.8	37500.3	56064.3	38414.9	29367.3
4349.75	2236.03	1553.6	1169.88	1910.44	3404.63	1765.69	1850.24	539.181	9236.33	4582.94	7216.36	12631	7851.18	7032.12	7519.08
73899.9	65724.2	64248	45767.3	45305.2	69409	65539.8	76862.7	33951.5	96154.4	103470	127531	131083	93665.7	79144.4	126919
4296.1	2292.33	4305.1		2462.43			549.298		7119.99	5892.8	4714.74	3540.48	8925.96	5607.45	3811.91
7902.83	7341.69	22433.5		4310.16	2915.98	3156.24	3463.78	6417.66	5713.76	5117.81	9707.88	2355.61	2043.51	4787.94	6078.4
16160	37930.6	31541	66163.2	85593	16894.5	32245.7	27799.1	33766	4657.77	2912.31	9481.02	4168.5	2114.86	6955.41	8660.14
266.277	856.281	571.88	506.926	376.802	364.845	682.634	452.334	215.552	58424.6	56043.2	93633.6	65083.1	102342	53663.6	44654.4
48953.5	29251.7	68420.3	24997.1	61619.8	17983.8	33391.7	25431.2	34244.1	219189	77326.6	118096	42936.3	41006.4	73788.5	79014.6
7948.75	2952.6	9545.52	10068.3	1232.61	12390.7	3080.01	3707.46	5161.26	392.543	16551.5	10798.8	8516.9	7380.28	6981.33	4213.48
3783.92	2489.53	5268.72	1265.4	1010.64	1924.57	3542.14	838.503	2230.31	1870.22	4703.28	4930.72	3237.85	3767.63	3760.62	2304.63
7474.86	15653.6	7812.81	3945.51	6757.07	6071.11	7135.52	9361.44	2744.27	18842.3	31282.8	31291.1	21727.6	31719.7	30523.2	26042.2
364806	225657	321363	191018	309803	239583	176509	206885	186269	360152	382435	391858	259546	290608	418804	298443
4032.01	979.873	4534.33	577.045	2150.59	2508.51	2226.86	2961.9	3851.01	8782.54	6098.64	7588.3	1666.23	2650.83	6037.19	4304.77
40642.1	50326.1	46462.8	30479.7	40096.4	23825.8	26021.1	26838.7	32352.2	53215.3	57264.6	73784.3	53159.5	53697.2	48173.4	39072.4

Figure 2. The order of the samples in the input tables should be aligned with the group variables. Here we see 5 Control, then 5 Treatment, and 5 Compound samples.

<u>Setting up the group_names and number of samples_per_group:</u>

Assuming another imaginary cell line experiment, the proteome of 10 Control samples, 12 Treatment samples, and 9 Compound samples has been quantified with MaxQuant.

Group names and the number of samples of each group are defined in the parameters *group_names* and *samples_per_group* respectively, and they are both entered as vectors [using the notation **c()**]:

```
ProtE_analyse(file = "C:\\Bioprojects\\BreastCancer\\Proteomics\\MaxQuant\\ProteinGroups.txt"
,group_names = c("Control", "Treatment", "Compound"), samples_per_group = c(10, 12, 9))
```

Note that *group_names* is a character vector and henceforth its elements are written within apostrophes, while *samples_per_group* is a numeric vector. The order of the *group_names* inside the vector must follow the order of the groups by which samples have been arranged in the input proteome table. In the input table of this example, Control samples have been arranged at the leftmost of the proteome table, Treatment samples at the middle, and Compound samples at the rightmost. Similarly to group_names, the order of the numbers in the *samples_per_group* vector must follow the ordering of the groups (10 Control, 12 Treatment, 9 Compound samples).

If ProteomeDiscoverer quantification was run *as batch*, multiple PD export files (corresponding to individual samples) would be created. In the absence of a metadata file, users will have to arrange the ProteomeDiscoverer exports in distinct folders, based on the experimental group they belong to. The folder names will be utilized as the exact group names, thus renaming the folders appropriately before feeding them to R, may be considered here. For example, assuming an imaginary experiment in which samples are organized into three groups (Control, Treatment, Compound), with each group containing 5 PD exports. The Figure 1 below shows the structure of the directories/folders:

```
C:\Bioprojects\BreastCancer\Proteomics\PD\
│
├── Control
│   ├── Sample1.xlsx
│   ├── Sample2.xlsx
│   ├── Sample3.xlsx
│   ├── Sample4.xlsx
│   └── Sample5.xlsx
│
├── Treatment
│   ├── Sample6.xlsx
│   ├── Sample7.xlsx
│   ├── Sample8.xlsx
│   ├── Sample9.xlsx
│   └── Sample10.xlsx
│
└── Compound
    ├── Sample11.xlsx
    ├── Sample12.xlsx
    ├── Sample13.xlsx
    ├── Sample14.xlsx
    └── Sample15.xlsx
```

Figure 3. The structure of folders, directories, and Excel files that can be input with *pd_multi*.

The input required in this case includes the file paths of the folders. These are parsed into the function by separating the folder names either with 2 backslashes (****) or via a single forward slash (**/**):

```
ProtE_analyse(pd_single_dir = c("C:\\Bioprojects\\BreastCancer\\Proteomics\\PD\\Control",
                                "C:\\Bioprojects\\BreastCancer\\Proteomics\\PD\\Treatment",
                                "C:\\Bioprojects\\BreastCancer\\Proteomics\\PD\\Compound"),
              normalization = "Cyclic_Loess", imputation = FALSE)
```

Output directory:

The output files are created inside a folder named ProtE_analysis followed by the data of the run and a number which corresponds to the number of the ProtE rans in that period. The folder will be located in the last directory of the path of the input file that the user has provided in the *file* or *pd_single_dir* parameters. It contains 3 subfolders Data_processing, Statistical_analysis, and Plots (**Figure 4**).

```
ProtE_Analysis/
├── Data_processing/
│   ├── Normalized.xlsx
│   ├── Dataset_Imputed.xlsx
│   └── Dataset_after_filtering.xlsx
├── Statistical_Analysis/
│   ├── Dataset_limma_test.xlsx
│   ├── Statistics.xlsx
│   ├── Sample_QC.xlsx
│   └── GSEA_results.xlsx
└── Plots/
    ├── Boxplot_before_processing.bmp
    ├── Violin_plot_before_processing.bmp
    ├── After_Processing_Boxplot.bmp
    ├── Kruskal_significant_heatmap.bmp
    ├── ControlvsTreatment_GSEA_plot.bmp
    ├── ControlvsTreatment_volcano.bmp
    ├── Proteins_abundance_rank.bmp
    ├── Unprocessed_meansdplot.bmp
    ├── Imputed_values_histogram.bmp
    ├── PCA_plot_allData.bmp
    └── normalized_meansdplot.bmp
```

Figure 4. The output directories of ProtE analysis folder.

ProtE pipeline

ProtE_analyse() follows a standardized Proteomics analysis workflow, which is first processing of the data, then proceeding to differential expression analysis and enrichment analysis, while also producing quality check metrics and plots.

The data are processed via:

1. Annotation fetching for the input files that do not include established Description.
2. Normalization of proteomic intensity values
3. Filtering based on the percentage of missing values of each protein.
4. Imputation of missing data to ensure robust downstream analysis.

It is important to note that ProtE works by processing and analyzing already existing protein quantification tables generated by popular spectral analysis tools (e.g., MaxQuant, ProteomeDiscoverer, DIA-NN). Therefore, ProtE cannot influence the upstream data processing decisions made by users within those tools. We strongly

recommend that users are familiar with the documentation and specific instructions for their chosen spectral analysis software and are aware of the importance of settings during the database search and the peptide-to-protein quantification.

Description fetching

If the descriptive information column is not available in the input files, annotation information about the Protein IDs, is important to be provided. For example, the input file: *.pg_matrix.tsv from DIA-NN usually does not provide this information. Detailed data is obtained from the UniProt database, using the *queryup* package. [1] The Description information will be shown in all Excel files created that contain the dataset and its statistics. It is usually present in the form of: **Acyl-coenzyme A thioesterase 1 OS=Homo sapiens OX=9606 GN=ACOT1 PE=1 SV=1 - [ACOT1_HUMAN]** for the ACOT1 protein.

Normalization

Mass Spectrometry quantitative data produced by software tools such as Proteome Discoverer and MaxQuant may be required to be processed with Normalization methods to reduce their systemic bias, which could be introduced due to technical or analytical reasons.[2] By normalizing the data, we aim to mitigate introduced technical variation and thus we enable more robust statistical calculations[3]. The ProtE package offers different methods of normalization that are provided via the *normalization* argument. As proteomics data is based on MS detected intensities, their values usually have high variation, compared to scaled expression matrices of other techniques.

These options include a simple **$\log_2$ transformation** of the data which aims to reduce the effect of proteins with the highest abundances. Two other methods included are **Quantile** and **Cyclic loess** Normalization, which are both applied to the $\log_2$ transformed data and are implemented using functions of the *limma* package[4]. Cyclic Loess normalization employs iterative loess regression (a technique that fits curves to nearby data points without assuming straight lines) on pairwise M-A plots (M: the log-ratio difference between samples; A: their average log-intensity), to reduce their dissimilarities [5]. Meanwhile, Quantile normalization assertively aligns empirical distributions to the data to make every feature's profile identical across samples [6]. Additionally, Variable Stabilizing Normalization (VSN) is also available implementing the corresponding function of the *limma* package[4] . In proteomics, proteins with high intensities often show bigger variances, so VSN normalizes the data to make up for this variability and thus helping spot true biological changes and reduce the extra noise of these proteins.

The data can also be transformed with **median normalization**, in which all intensity values are divided by each sample’s median intensity, resulting in a median equal to 1 across the protein data set [2]. Other normalization methods share an initial step of dividing each intensity value by the total sum of intensities for its respective sample. This converts absolute intensities into relative proportions of the total intensity, effectively scaling samples to a common baseline. For instance, if technical variations, like uneven protein loading, cause one sample's overall intensities to be twice as high as another's, this step equalizes the baselines, preventing technical-derived high fold changes and allowing robust cross-sample comparisons of biological signals. After dividing the

intensities by the sum, **Total Ion Current normalization** then rescales the values by multiplying them by the average expression of the protein, while **Parts Per Million (PPM) normalization** scales the values by multiplying them by one million[7].

Of note, as the data output from DIA-NN has already been normalized with the MaxLFQ quantification[8], any additional normalizing methods should be be applied cautiously, within the dianno function. The same applies to MaxQuant output that includes the already normalized data in LFQ intensity columns.

**Normalization Method**	**Argument Value**	**Description**	**Notes**
Log2 Transformation	log2	Applies a simple log2 transformation to the data.	Basic transformation, no additional steps.
Cyclic Loess Normalization	Cyclic_Loess	Reduces dissimilarities using Cyclic Loess normalization.	Applied after log2 transformation; uses limma package functions.
Quantile Normalization	Quantile	Makes the distribution of each feature identical.	Applied after log2 transformation; uses limma package functions.
Median Normalization	median	Divides intensity values by each sample's median intensity (median = 1).	Ensures median intensity is 1 across the dataset.
Total Ion Current (TIC)	TIC	Divides intensities by total sum, rescales by average intensity of each protein.	Used to limit technical variability by reducing the effect of samples with higher sum intensities
Parts Per Million (PPM)	PPM	Divides intensities by	Used to limit technical variability by reducing the

**Normalization Method**	**Argument Value**	**Description**	**Notes**
		total sum, scales by 1 million.	effect of samples with higher sum intensities. Normalizes in a way to have a fixed scaling factor
Variable Stabilizing Normalization (VSN)	VSN	Applies Variable Stabilizing Normalization.	Uses limma package functions. Reduces the additional variance of high intensity proteins.
None (for DIA-NN/MaxQuant)	FALSE	No additional normalization (data already normalized).	Is the default method when normalization is undefined or not desired

Table 2. Information about the normalization methods that can be selected with function ProtE_analyse()

Filtering of Missing Values

When working with mass spectrometry-based proteomics data, a common issue encountered is the presence of missing values in the intensity measurements for each protein. These missing values can occur for several reasons, technical, analytical and biological. Some proteins may not be identified in the sample due to technical limitations, their abundances may fall below the detection limit of the analyzing instrument, or the proteins may be completely absent from the examined sample.[9]. The fact that each sample is quantified in unique spectral processing rans, also introduces variation in the chance of identifying proteins of low frequency.

In proteomics statistical analysis, the presence of missing values in protein abundances can alter the statistical robustness of the implicated tests. That is why excluding the proteins with high amounts of missing values, called filtering, is a common practice in proteomics pipelines.

ProtE offers the option of filtering Proteins based on the percentage of missing values they contain. Specifically, functions include the argument *filtering_value*, which refers to the percentage of missing values per protein allowed to remain in the filtered dataset. If the user sets it to 100, no filtering will occur, and proteins will not be excluded based on their frequency of detection and quantification. However, if the user sets it to 0, only the proteins with no missing values in all samples will be kept. In metabolomics analyses, a common threshold is defined by the 80% rule, where only metabolites present in 80% percent of the samples are kept[11]. That means that if we followed an identical rule, we

would have to set the *filtering_value* to 20, to keep proteins with maximum 20% percent missing intensities in all samples. However, in proteomics studies no globally accepted threshold is used. By selecting a high *filtering_value* noise can be introduced due to increased missing values, while selecting a low filtering_value can lead to loss of information and vice versa[13]. The choice remains in the user's hands.

The parameter *global_filtering* determines if filtering for missing values will be performed across all groups or separately inside each group. That is important, in case the user wants to keep proteins with high missing values, that could be influenced by biological parameters. Specifically, if a protein is only identified in one group of comparison and does not appear in the other groups, it would have a high percentage of missing values across all samples; but that could be explained by biological reasons, as the protein would truly have no expression in one group (e.g., Disease vs Control). By setting *global_filtering* to FALSE, the filtering is less strict to allow such proteins, to remain in the dataset.

Also, the reverse positive proteins (REV) will be excluded when the input is the ProteinGroups.txt from MaxQuant.

Imputation

The ProtE package also offers a few options for estimating the missing values, via the argument *imputation* of each function. Specifically, the remaining missing values after the filtering process are substituted by predicted quantitative values. The goal is to replace missing values arising from technical limitations with biologically relevant estimates, thereby reducing bias and enhancing the robustness of downstream statistical analyses[15].

The available imputation methods are split into categories of simple deterministic values, stochastic distribution-based values and model-based multivariate values. The first category includes simply assigning the **limit of detection ("LOD")** of the experiment (lowest abundance value in the dataset), or **its half (LOD/2)** to the missing values or assigning **the mean abundance ("mean")** of each protein to the missing values. Additionally, imputation of the missing values **by simply assigning 0 values to them is possible ("zeros").** The stochastic distribution-based methods derive from the mentioned ones above, as the first imputes with **values derived from the Gaussian distribution centered on the LOD ("Gaussian_LOD"**) and **sampling from a Gaussian distribution around the mean abundance of each protein ("Gaussian_mean_sd")** [10]. All these methods are the ones mostly used in proteomics experiments [10] and are characterized by the low computation resources need and their quickness. The LOD and zero used methods assume that missingness is a result of biological and analytical reasons, while mean replacement methods assume that they are a result of technical limitations.

The other 2 imputation methods are based on other well-defined R packages, based on machine learning models. Specifically, **k-nearest neighbors ("kNN")** imputation is available from the package VIM[12] and "**missRanger"**, a quicker multivariate imputation algorithm alternative to missForest (based on random forests), from the self-titled R

package [14]. These 2 imputation algorithms are better in predicting missingness stemmed from both technical and biological reasons[19], however they require more computational resources and are time costly.

The processing results of each data processing step (e.g., firstly the normalized data, then the filtered, and finally the imputed dataset) are displayed in Excel files inside the Data_processing folder. The power of ProtE lies in its ability to run on different processing settings, by simply changing the functions' parameters. The new results will be saved in a new ProtE_analysis_date_number folder in the same directory, allowing the user to compare the results of the different processing steps, and picking what fits best to their data.

**Imputation Method**	**Argument Value**	**Description**	**Notes**
Limit of Detection (LOD)	LOD	Assigns the lowest abundance value in the dataset to missing values.	Reflects the experiment's detection limit.
Half of Limit of Detection	LOD/2	Assigns half of the lowest abundance value to missing values.	A more conservative imputation than LOD.
Gaussian LOD	Gaussian_LOD	Assigns values derived from a Gaussian distribution of the LOD to missing values.	Introduces variability around the LOD.
Missing Values as Zeros	zeros	Treats missing values as zeros.	Assumes missingness indicates no detection.
Mean Abundance	mean	Assigns the mean abundance of each protein to its missing values.	Assumes missingness is random and uses protein mean.
Gaussian Mean and SD	Gaussian_mean _sd	Samples from a Gaussian distribution around the mean abundance of each protein.	Adds variability around the mean for missing values.

ProtE

Imputation Method	Argument Value	Description	Notes
k-Nearest Neighbors (kNN)	kNN	Imputes missing values using the k-nearest neighbors algorithm.	Implemented via the VIM package [10].
missRanger	missRanger	Uses a multivariate imputation algorithm based on random forests.	Faster alternative to missForest; uses missRanger pa ckage [11].
No Imputation	FALSE	No imputation is applied to missing values.	Default if imputation is not desired.

Table 3. Information about the imputation methods that can be selected with function ProtE_analyse() The results of each data processing step (e.g., the normalized then filtered, and finally imputed dataset) are displayed in Excel files inside the Data_processing folder. Note that the Imputed_data excel file will include data which will have already been normalized.

Statistical analysis

After the proteomics data are processed, ProtE functions perform downstream statistical analysis, to determine proteins with significantly different abundances between the experimental groups of comparison and a variety of quality checks. The analysis is conducted between the distinct groups and their samples. Using the *independent* argument, the user can choose whether the group variables should be analyzed as independent when *independent* = TRUE (e.g. Control vs Patients) or as matched when *independent* = FALSE (e.g. patients Before and After treatment, or cell lines across different times of drug induction). When the analysis is between paired groups, the number of samples in each one of them must be identical, and they should be ordered based and the subject they derive from. So, in the proteomics data table or the metadata table of a paired group of 5 samples across 2 timepoints, the first column or row respectively of each group should be derived from the same subject, the same for the second, the third and so on.

The Excel file traditional_statistics.xlsx on the output folder Statistical_analysis encapsulates non-parametrical and traditional statistical tests' results, alongside valuable information for each gene. Specifically, it includes the average abundance and standard deviation of every gene per group, along with the ratios and the $\log_2$ fold changes of the averages between the groups. Depending on the selection of either TRUE or FALSE for the *independent* parameter, pairwise comparisons are conducted using the Mann-Whitney or the Wilcoxon signed-rank test, while for more than two groups the Kruskal Wallis or Friedman tests are also conducted. In the output, the p-values and adjusted p-values, for these tests are also featured. The user has the option of the p adjustment method that wants to be implemented via the *p.adjust.method* parameter. Extra

information includes homoscedasticity checks for each gene with the Bartlett and Levene tests p values being shown, as well as the multivariate pseudo-F , R^2 and p values from the univariate PERMANOVA test, utilizing the *adonis* test from vegan package[16].

Parametrical tests are performed utilizing the package *limma*, as it is commonly used for differential expression analysis in large omics datasets [4,17] and are saved in the Excel file limma_statistics.xlsx . Limma employes generalized linear model algorithms, to assess the differential expression across multiple features, even if their distribution is not normal. Specifically, the data undergoes log_2 transformation unless this step has already been performed in the normalization process. A linear model is fitted using a design matrix that includes the experimental groups and any covariates (included in the metadata file), followed by Empirical Bayes moderation, a standard approach in limma analyses. The model calculates coefficients for each experimental group in the design matrix, which represents their effects. When a *metadata_file* with covariates is provided, the covariate coefficients are included in the output, and these covariates are accounted for in the differential expression analysis. Additionally, if the *independent* parameter is set to FALSE, subjects with repeated measurements are treated as a random effect. An ANOVA-like F-test is performed testing just the experimental groups of comparison, when they are more than 2, while for every pairwise comparison a moderated t-test is computed. The output includes p-values, adjusted p-values, t-scores, and F-statistics from the Empirical Bayes moderated t-test and ANOVA-like test, alongside the averages, and log_2 fold changes for each gene across groups (based on the limma-processed data) and are presented in the Excel file.

The Excel file "Sample_QC.xlsx" contains information about the proteins present in each sample and the missing values before and after filtering. It also includes the scores of the first two principal components for the entire proteome.

Enrichment analysis

ProtE performs gene set enrichment analysis, to determine which gene sets are more associated with the significantly differentiated proteins. This analysis is conducted using as metric score the log_2 fold changes of the pairwise comparisons and a fast Gene Set Enrichment analysis (GSEA) is conducted, by implementing the package *fgsea [18]*. The user can select via the *species* parameter, for which species the analysis will be performed, and via the *subcollection* parameter from which collection will the pathways/gene sets be used (REACTOME, Hallmark or GO databases) . The results are saved inside an excel file named GSEA_results.xlsx that contains the enriched pathways of each comparison in different excel sheets.

Information about each argument is available in the help file that you can retrieve by writing ?ProtE_analyse in your console.

Visualization plots

To complement the Excel files with the results of data processing and the statistical analysis ProtE produces numerous plots, to interpret the data and provide the user with credible information. Here we will present examples of these plots that were created when we input jittered data into the functions.

Firstly, a boxplot and violin plot alongside mean vs standard deviation plots are produced before and after the processing of the data. Thus, they highlight the distribution and the variance of the values in each sample as well as how they were affected by the normalization and the imputation. They serve as a quick quality check: for instance, samples with unusually low median abundances in a boxplot may indicate technical issues in sample preparation or spectral identification, prompting removal from the statistical analysis.

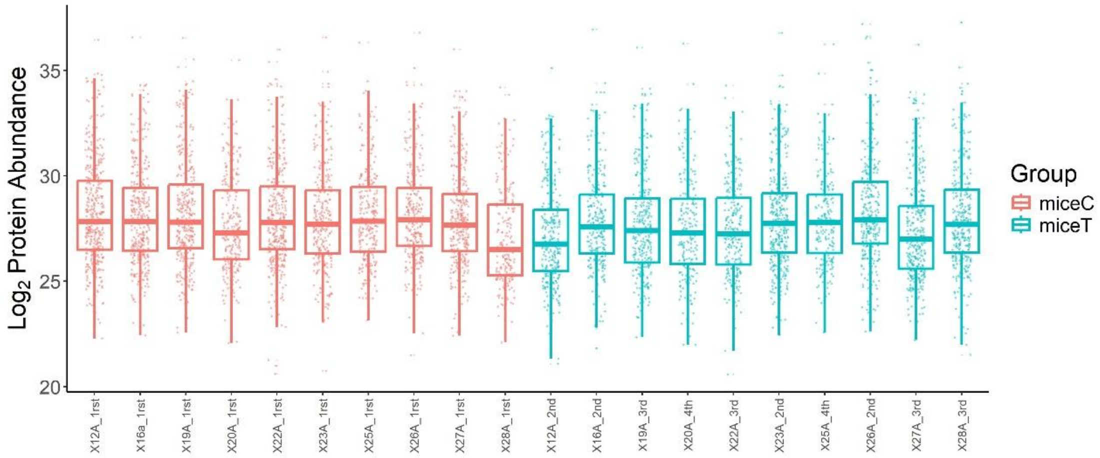

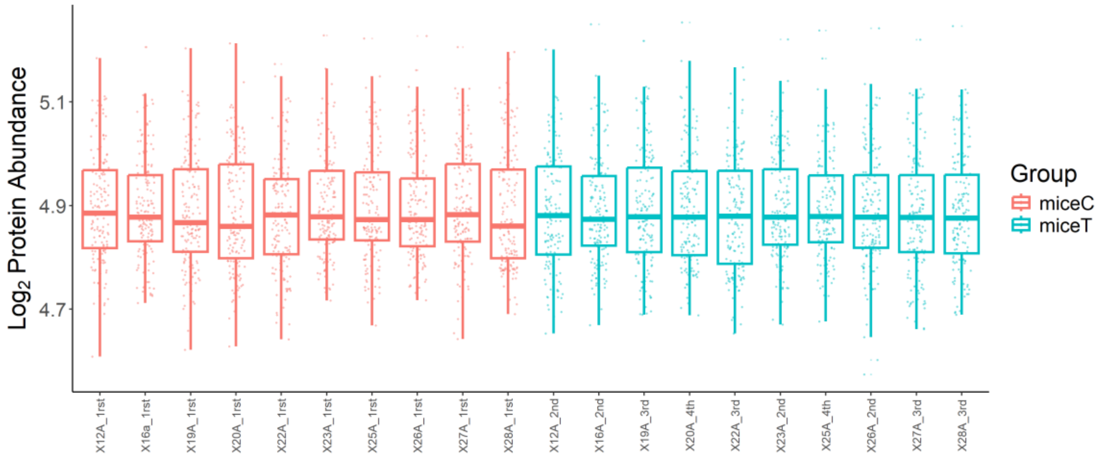

Figure 5. The boxplot of the data before (up) and after (down) processing.

The Coefficient of Variation is the ratio of the standard deviation to mean abundance of every protein. The plot is created prior to the processing of the data and gives an overview of the quantified protein measurements in each group. Lower CoV indicates higher precision and consistency in measurements within that group.

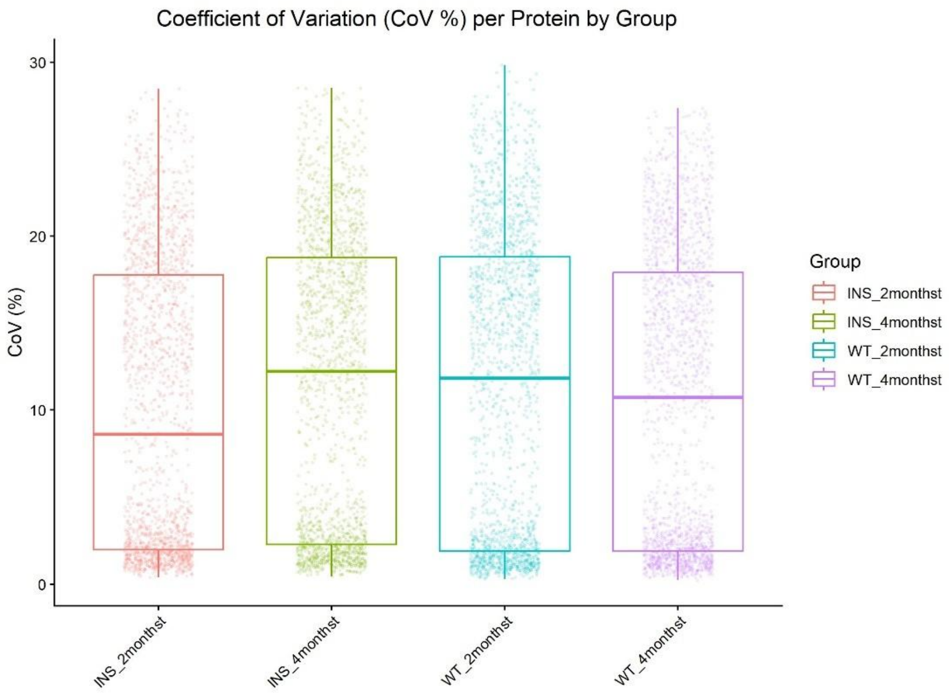

Figure 6. Coefficient of Variation (CoV%) per protein by Groups of comparison.

The mean vs SD plot showcases whether increased mean expression of the data correlates with increased standard deviation, which can be seen by an upward slope of the red line. If that is the case in the unprocessed data, a normalization method like vsn

would help, reducing this technical batch effect which would appear with a reduced slope of the red line.

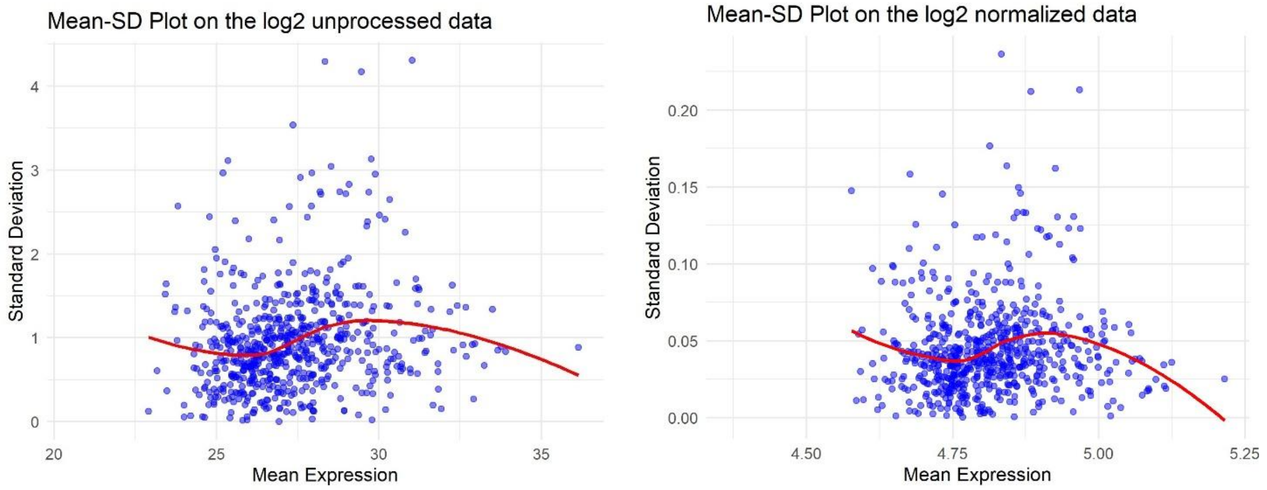

Figure 6. The mean sd plots before (left) and after (right) the processing of proteomics data with *vsn* normalization and *missRanger* imputation.

Additionally, a protein-ranked log2 abundance plot is provided, which also shows the percentage of missing values for each protein. It is informative about the distribution of Missing Values along the ranked proteins. If proteins with the highest abundances were of yellow color (e.g. high number of missing values), that could be a reason to apply a stricter filtering threshold, in the next run.

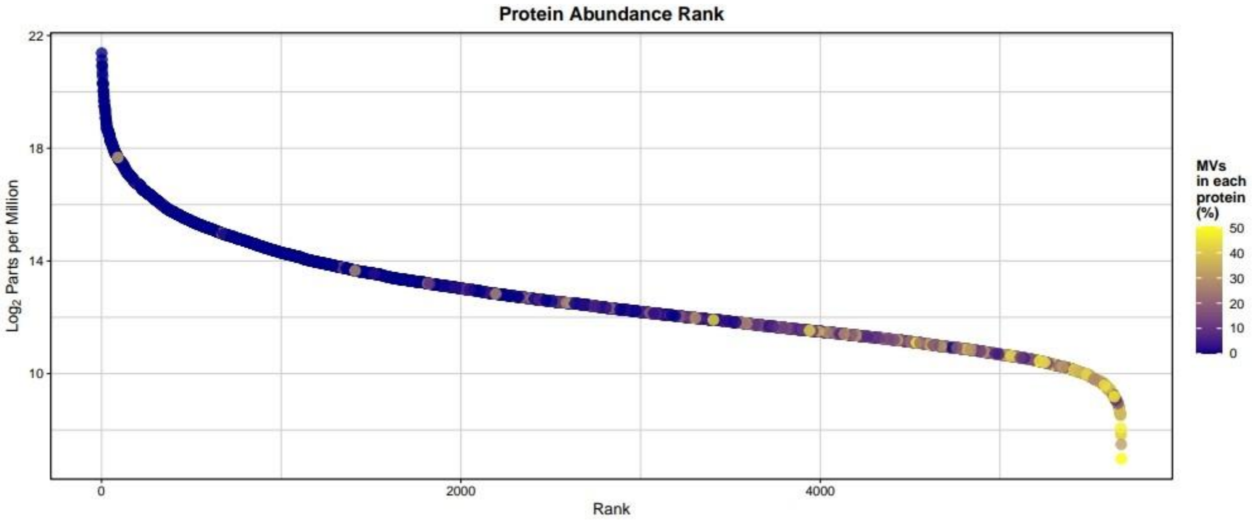

Figure 7. Protein Abundance Rank plot with information regarding the percentage of MVs of each feature.

When imputation methods *mean*, *kNN*, *Gaussian_mean_sd*, *Gaussian_LOD* and *missRanger* have been parsed, a histogram with the distribution of the imputed values and the initial ones, is created to inform the user about the imputation quality, and the distribution of the imputed values.

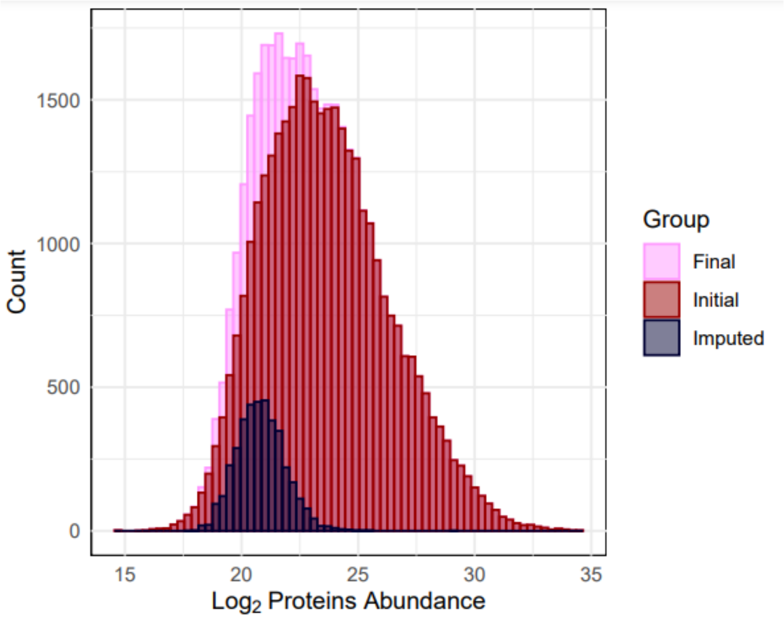

Figure 8. Histogram of the distribution of imputed values, available for multiple values replacement options.

Users can select the statistical method for determining significance by the logical setting *parametric* parameter. Setting *parametric = TRUE* uses results from the limma t-test or ANOVA-like F-test , while *parametric = FALSE* uses results from the Wilcoxon test or Kruskal-Walli's test. The user can further specify the significance threshold with the *significance* argument: setting *significance = "p"* uses a raw p-value threshold of 0.05, and *significance = "p.adj"* uses the selector's adjusted p-value threshold of 0.05.

Principal Component Analysis (PCA) is also performed on the log_2-transformed protein abundance data across samples. The data are scaled and centered before the analysis. To visualize its results, a PCA plot is created that displays all samples in a two-dimensional space, where the axes represent the first and second principal components. PCA is conducted to reduce the dimensionality of the data by projecting the samples into a 2D space, thereby enabling the detection of sample clustering, batch effects, or outliers. If the experimental groups of comparison appear separated in the PCA of the complete set of proteins, this means that the groups' proteome abundance profiles form biologically distinct clusters.

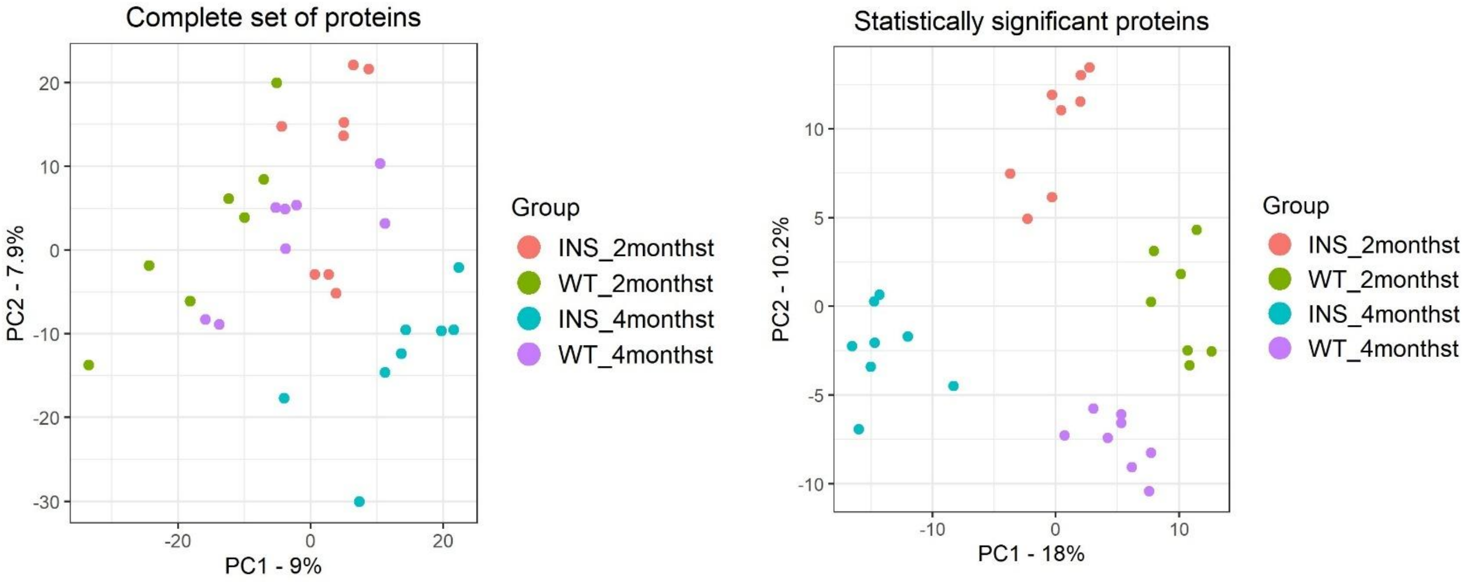

Figure 9. PCA plot derived from all the proteins (left) and the significant ones determined by the Kruskal Wallis test (right)

For experiments with just 2 groups a heatmap is produced showing the significant proteins of the selected test (via the *parametric* argument).It is split into the selected groups and created with the z-scale normalized abundances of each sample. In the heatmap clustering of the proteins in rows takes place. When there are more than 2 groups, one heatmap for the groups altogether is created by using the significant proteins of the F-test or Kruskal-Walli's/Friedman tests, alongside a heatmap for each pairwise comparison.

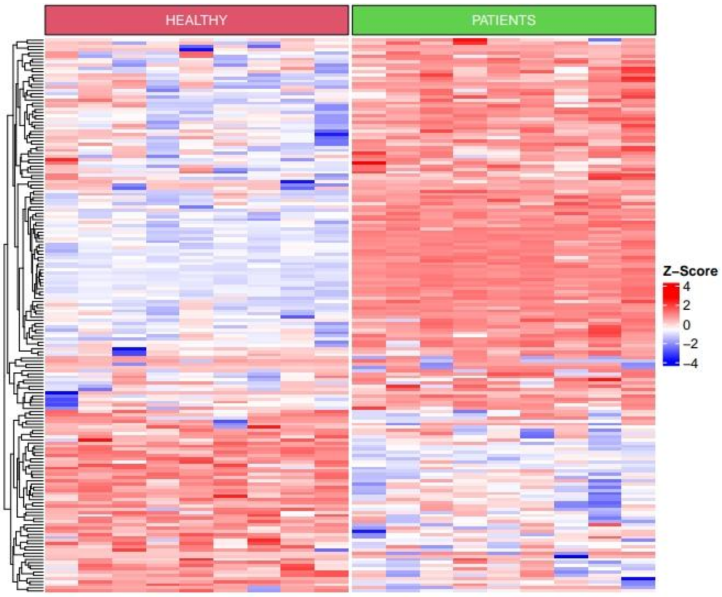

Figure 10. A heatmap for each pairwise comparison is created, while clustering of the proteins in rows takes place.

Additionally, Volcano plots are also being created for every pairwise comparison. With the *LFC* parameter the user can select the threshold of the Log_2Fold change from which the significant proteins (either with p or adjusted p value < 0.05) will be colored as up and downregulated.

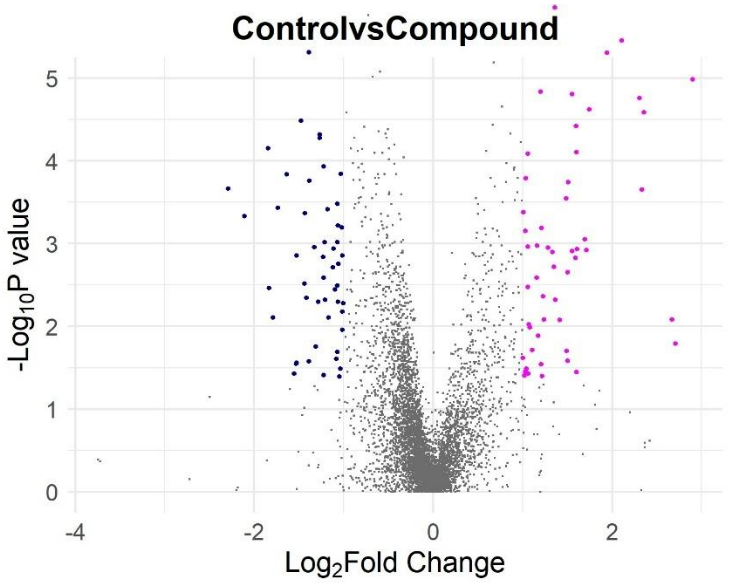

Figure 11. An example of a volcano plot that is created. In this example, the p value is selected as the Y-axis instead of the adjusted p value, and LFC threshold was set to 1.25. Lastly, to visualize the results of the GSEA, enrichment plots are created that showcase the significantly (p.adjusted < 0.05) down/upregulated gene sets between each comparison. The max number of pathways/ gene sets in both up and downregulation are 7. If the significant ones are more than 7, the 7 with the highest NES score are selected.

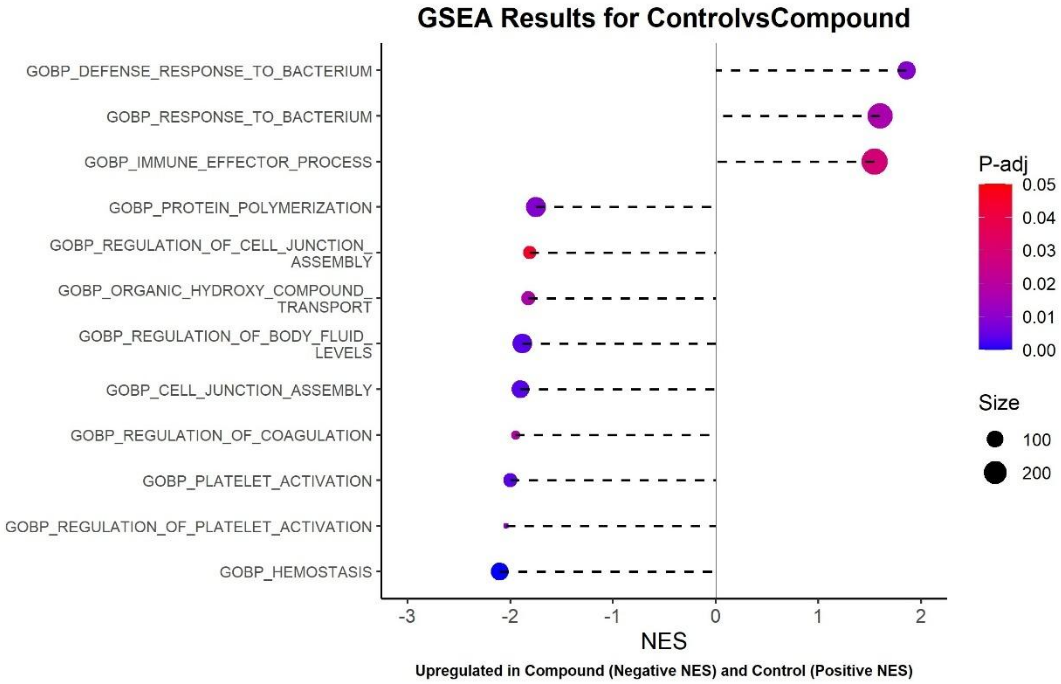

Figure 12. The enrichment plot showcasing the results of the GSEA.

All visualization plots are saved as BMP files and are inside the Plots subfolder of the ProtE_analysis directory.

References

[1] Voisinne G, queryup: Query the UniProt REST API using R. 2019.

[2] Välikangas, T., Suomi, T., Elo, L.L., A systematic evaluation of normalization methods in quantitative label-free proteomics. *Brief Bioinform* 2018, 19, 1–11.

[3] Välikangas, T., Suomi, T., Elo, L.L., A systematic evaluation of normalization methods in quantitative label-free proteomics. *Brief Bioinform* 2018, 19, 1–11.

[4] Ritchie, M.E., Phipson, B., Wu, D., Hu, Y., et al., limma powers differential expression analyses for RNA-sequencing and microarray studies. *Nucleic Acids Res* 2015, 43, e47.

[5] Bolstad, B.M., Irizarry, R.A., Astrand, M., Speed, T.P., A comparison of normalization methods for high density oligonucleotide array data based on variance and bias. *Bioinformatics* 2003, 19, 185–93.

[6] Zhao, Y., Wong, L., Goh, W.W. Bin, How to do quantile normalization correctly for gene expression data analyses. *Sci Rep* 2020, 10, 15534.

[7] Wulff, J.E., Mitchell, M.W., A Comparison of Various Normalization Methods for LC/MS Metabolomics Data. *Advances in Bioscience and Biotechnology* 2018, 09, 339–351.

[8] Cox, J., Hein, M.Y., Luber, C.A., Paron, I., et al., Accurate proteome-wide label-free quantification by delayed normalization and maximal peptide ratio extraction, termed MaxLFQ. *Mol Cell Proteomics* 2014, 13, 2513–26.

[9] Karpievitch, Y. V, Dabney, A.R., Smith, R.D., Normalization and missing value imputation for label-free LC-MS analysis. *BMC Bioinformatics* 2012, 13 Suppl 16, S5.

[10] Harris, L., Fondrie, W.E., Oh, S., Noble, W.S., Evaluating Proteomics Imputation Methods with Improved Criteria. *J Proteome Res* 2023, 22, 3427–3438.

[11] McGurk, K.A., Dagliati, A., Chiasserini, D., Lee, D., et al., The use of missing values in proteomic data-independent acquisition mass spectrometry to enable disease activity discrimination. *Bioinformatics* 2020, 36, 2217–2223.

[12] Kowarik, A., Templ, M., Imputation with the *R* Package **VIM**. *J Stat Softw* 2016, 74.

[13] Arioli, A., Dagliati, A., Geary, B., Peek, N., et al., OptiMissP: A dashboard to assess missingness in proteomic data-independent acquisition mass spectrometry. *PLoS One* 2021, 16, e0249771.

[14] Mayer M., missRanger: Fast imputation of missing values. R package version, 2(0). 2019.

[15] Kong, W., Hui, H.W.H., Peng, H., Goh, W.W. Bin, Dealing with missing values in proteomics data. *Proteomics* 2022, 22.

[16] Oksanen J, S.G.B.F.K.R.L.P.M.P.O.R.S.P.S.M.S.E.W.H.B.M.B.M.B.B.B.D.B.T.C.G.C.M.D.C.M.D.S.E.H.F.R., vegan: Community Ecology Package. *R package version 2.7-0* 2024.

[17] Hutchings, C., Dawson, C.S., Krueger, T., Lilley, K.S., Breckels, L.M., A Bioconductor workflow for processing, evaluating, and interpreting expression proteomics data. *F1000Res* 2023, 12, 1402.

[18] Korotkevich, G., Sukhov, V., Budin, N., Shpak, B., et al., Fast gene set enrichment analysis. *bioRxiv* 2016.

[19] Hamid, Z., Zimmerman, K.D., Guillen-Ahlers, H., Li, C., et al., Assessment of label-free quantification and missing value imputation for proteomics in non-human primates. *BMC Genomics* 2022, 23, 496.
